# COVID-19-induced silent myocarditis and newly developed hypertension in a 3-year-old boy

**DOI:** 10.1186/s43044-022-00282-w

**Published:** 2022-05-31

**Authors:** Elaheh Malakan Rad, Sara Momtazmanesh

**Affiliations:** 1 Department of Pediatric Cardiology, Children’s Medical Center (Pediatric Center of Excellence), Tehran University of Medical Sciences, Tehran, Iran; 2 School of Medicine, Tehran University of Medical Sciences, Tehran, Iran

**Keywords:** COVID-19, Myocarditis, Dilated cardiomyopathy, Strain imaging, Speckle-tracking, Echocardiography, Magnetic resonance imaging, Hypertension, Case report

## Abstract

**Background:**

COVID-19 myocarditis occurs in 7–28% of patients admitted in the hospital with or without multisystem inflammatory syndrome. It may present as fulminant myocarditis. Dilated cardiomyopathy as a sequela of COVID-19 myocarditis has been reported in the pediatric population. However, to date, no case of silent COVID-19 myocarditis progressing to dilated cardiomyopathy has been reported in children. Furthermore, although newly developed hypertension as a sequela of COVID-19 infection has been reported in adults, there is no report of newly developed COVID-induced hypertension in children. We report a 3-year-old boy with silent COVID-19 myocarditis progressing to dilated cardiomyopathy and newly developed systemic hypertension.

**Case presentation:**

A 3-year-old boy was referred to the emergency department because of respiratory distress. The parents gave a history of SARS-CoV-2 infection in the child 5 months ago that was manifested as fever and cough, for which he was treated as an outpatient. Echocardiographic examination revealed a severe decrease in left ventricular systolic function in favor of dilated cardiomyopathy. Cardiac magnetic resonance imaging established the diagnosis of myocarditis. The patient left ventricular systolic function did not improve after 2 weeks of intravenous inotropic support. Therefore, the child was transferred to another tertiary center with extracorporeal membrane oxygenation and pediatric cardiac transplantation facilities.

**Conclusions:**

COVID-19 can induce silent myocarditis with progression to dilated cardiomyopathy and newly developed systemic hypertension. Thus, a thorough examination of the heart and measurement of blood pressure are mandatory in every child with COVID-19 infection. Cardiac MR is an indispensable tool in the diagnosis, follow-up, and prognostication of COVID-19 myocarditis. Moreover, four-chamber speckle tracking strain imaging showed apical rocking in all the four heart chambers in this child with opposite direction in the failed left ventricle compared with other cardiac chambers. Lastly, the presence of septal flash on M-mode echocardiography, apical rocking and prestretch–rebound stretch patterns on longitudinal strain imaging of the failed left ventricle in this child may be of predictive value for response to cardiac resynchronization therapy.

**Supplementary Information:**

The online version contains supplementary material available at 10.1186/s43044-022-00282-w.

## Background

COVID-19 myocarditis occurs in 7–28% of patients admitted in the hospital with or without multisystem inflammatory syndrome (MIS) [1]. It may present as fulminant myocarditis [2]. Dilated cardiomyopathy as a sequela of COVID-19 myocarditis has been reported in the pediatric population [3]. However, to date, no case of silent COVID-19 myocarditis progressing to dilated cardiomyopathy has been reported in children. Furthermore, although newly developed hypertension as a sequela of COVID-19 infection has been reported in adults, there is no report of newly developed COVID-induced hypertension in children [4]. We report a 3-year-old boy with silent COVID-19 myocarditis progressing to dilated cardiomyopathy and newly developed systemic hypertension. We also present the findings on speckle-tracking strain imaging echocardiography of the four chambers of the heart in this patient.

## Case presentation

A three-year-old was referred to the emergency department because of respiratory distress. He was a well-nourished boy with normal temperature and a respective heart rate and respiratory rate of 140 beats and 63 per minute on physical examination. His blood pressure was 135/110 mmHg. Cardiac auscultation revealed muffled heart sounds with a grade 2/6 regurgitant systolic murmur at the cardiac apex. The rest of the physical examination was unremarkable. The parents gave a history of SARS-CoV-2 infection in the child five months ago that was manifested as fever and cough, for which he was treated as an outpatient. Furthermore, he had a history of complete cardiac evaluation for a cardiac murmur, including a comprehensive echocardiographic examination by a pediatric cardiologist one year ago, which confirmed a normal structure and function and the innocent nature of the murmur. Family history was negative except a COVID-19 infection in five months ago in other family members.

**Fig. 1:**
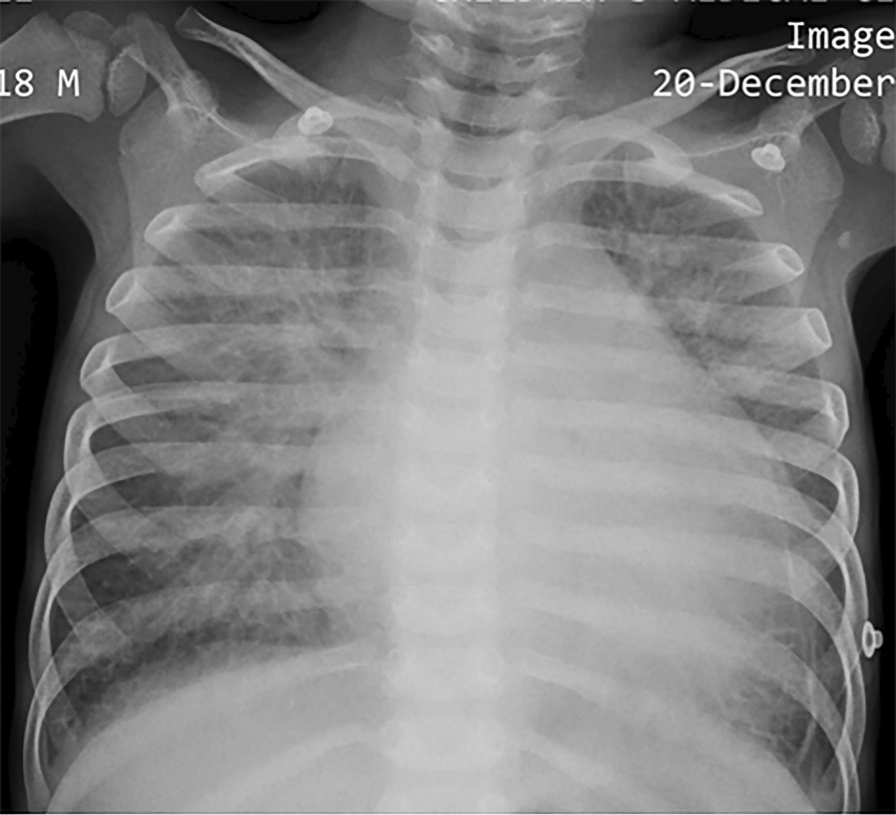
Chest X-ray of the patient showing cardiomegaly and pulmonary congestion

**Fig. 2:**
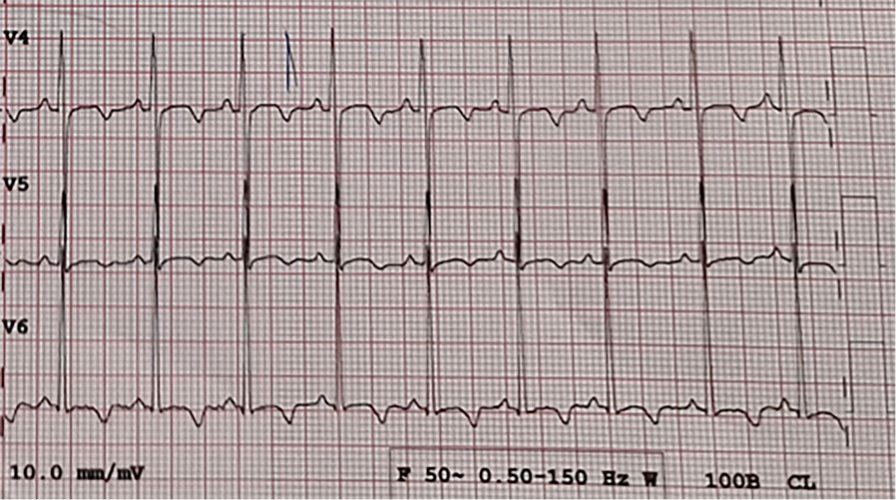
T wave inversion in left precordial leads of the electrocardiogram of the patient

Cardiomegaly, pulmonary edema, and blunted costophrenic angles were evident on the chest X-ray (Fig. 1). The electrocardiogram showed T wave inversion in left precordial leads (Fig. 2).

**Fig. 3:**
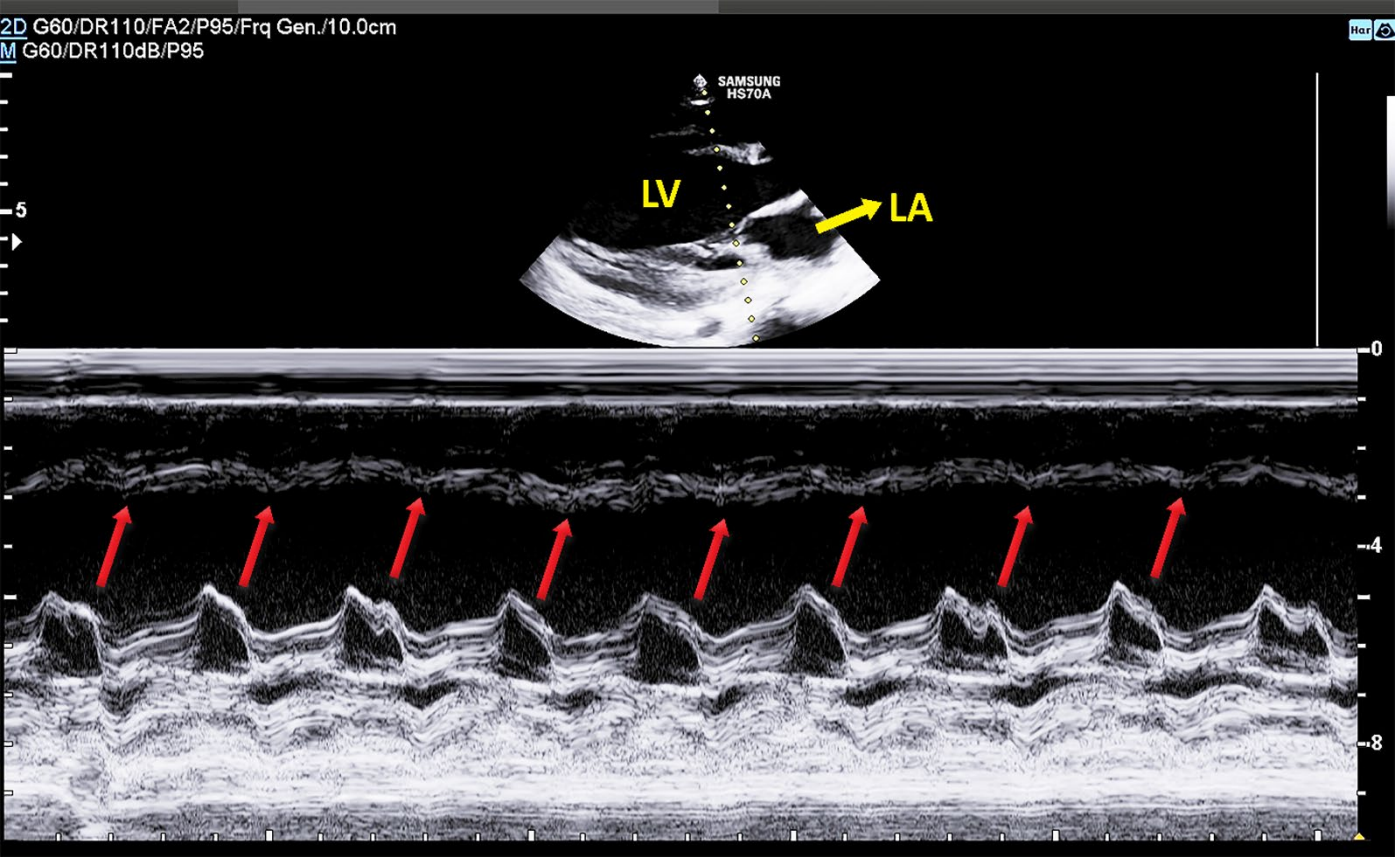
Septal flash on M-mode echocardiography of the patient

Comprehensive two-dimensional color Doppler echocardiography revealed a severe decrease in left ventricular systolic function in favor of dilated cardiomyopathy, significant left ventricular enlargement, mild to moderate holosystolic mitral regurgitation with eccentric posterolateral jet, trivial aortic regurgitation, and septal flash (Fig. 3). Left ventricular ejection fraction by Simpson's method was 26%, and tricuspid annular plane systolic excursion (TAPSE) was 21.8 mm. There was no coarctation of the aorta.

**Fig. 4:**
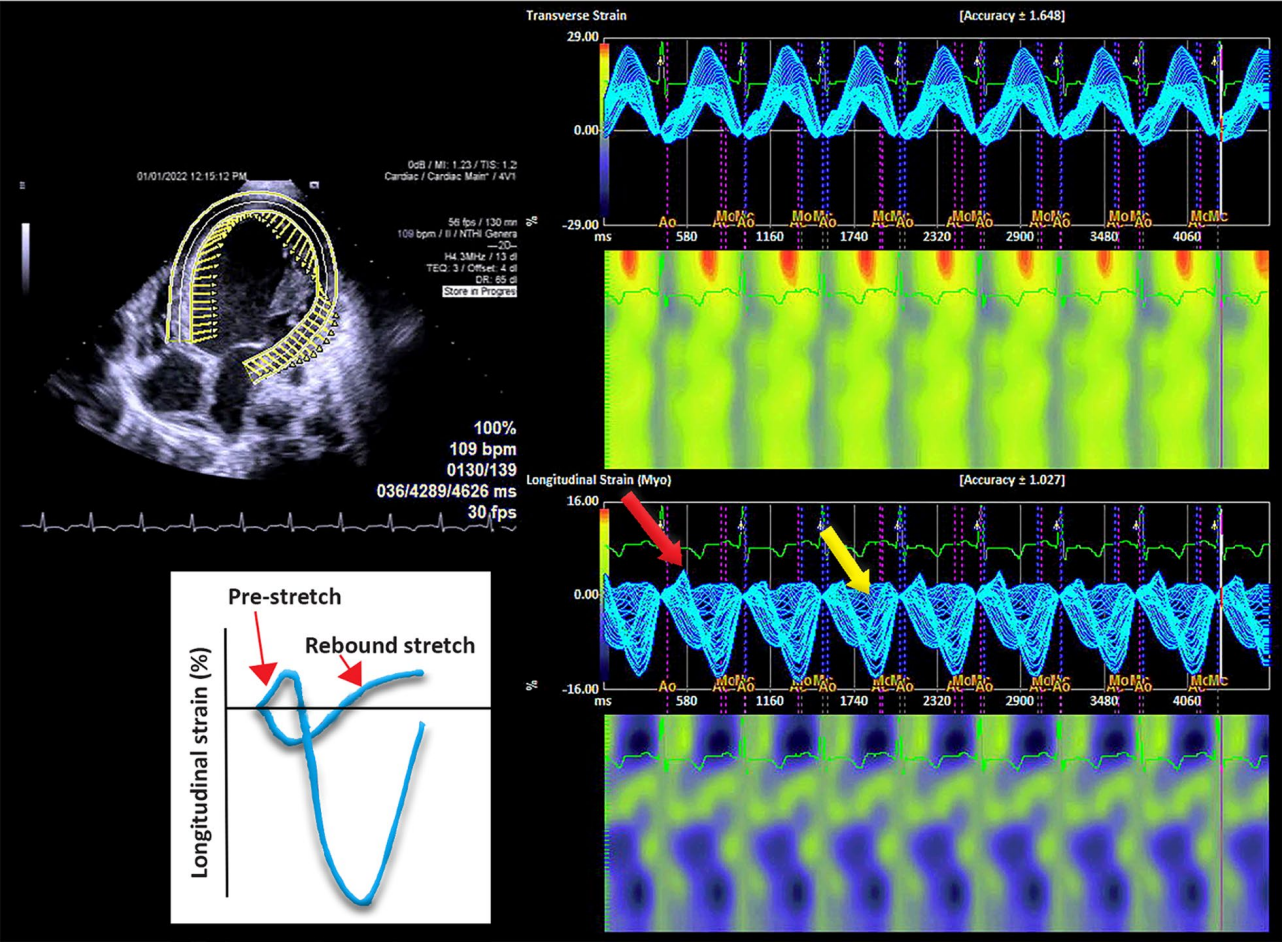
Left ventricular transverse and longitudinal strain imaging of the patient

**Fig. 5:**
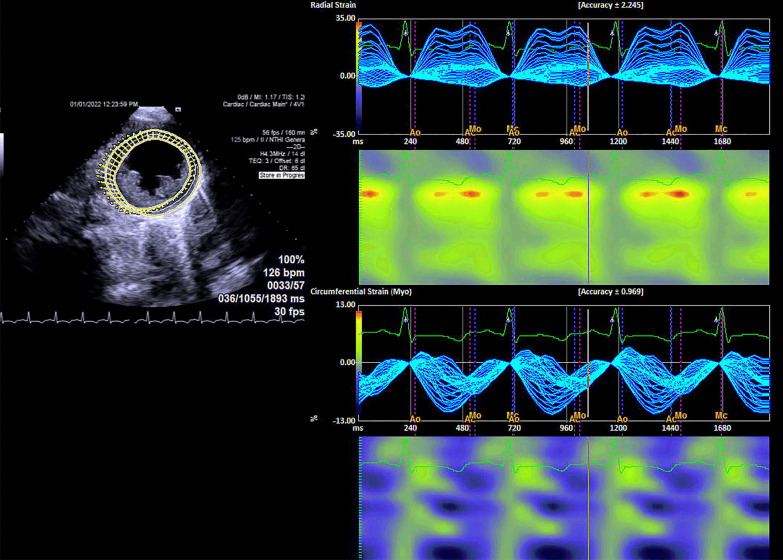
Left ventricular radial and circumferential strain imaging of the patient

**Fig. 6:**
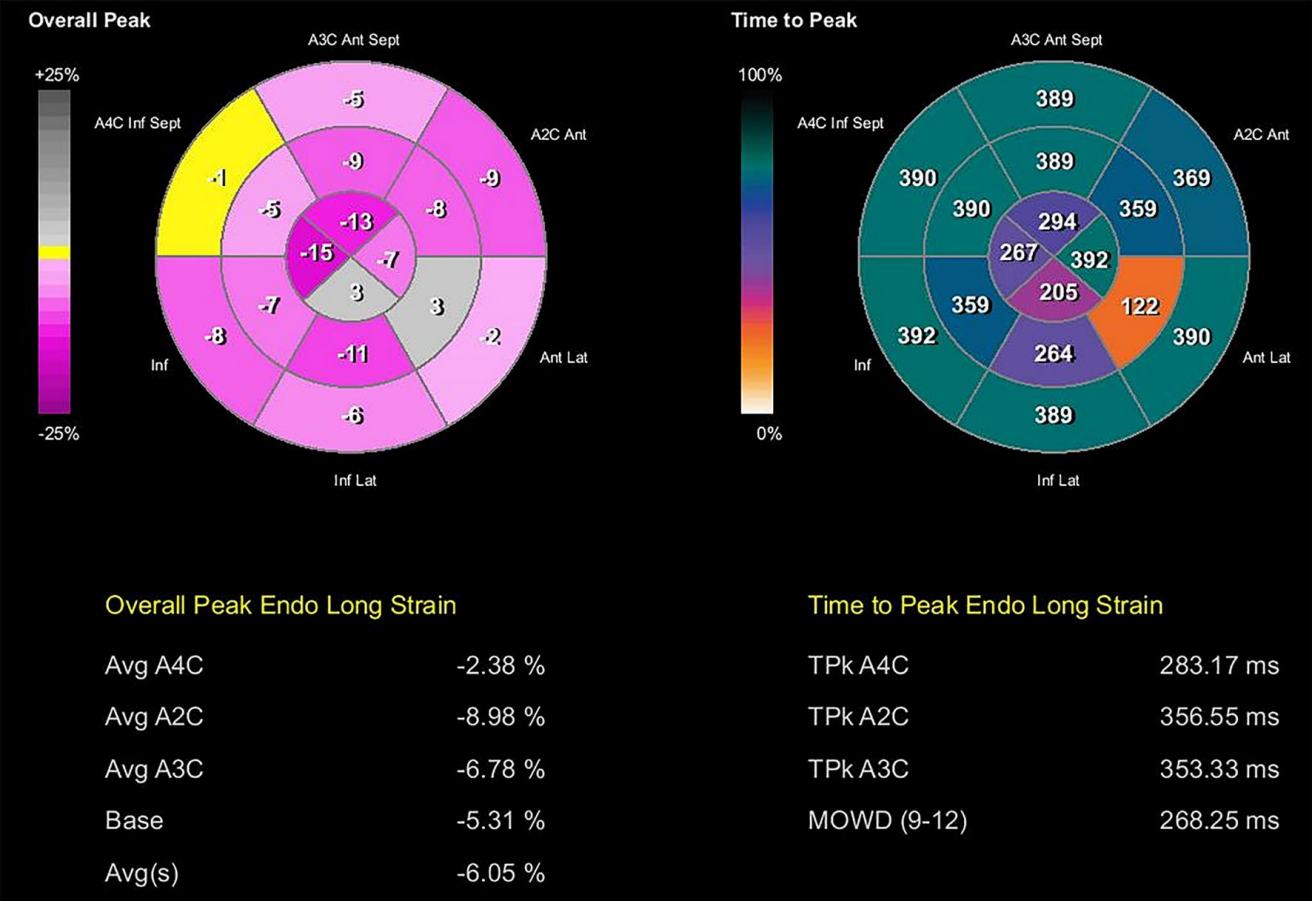
Bull’s eye plot of left ventricular longitudinal strain and time to peak longitudinal strain

**Fig. 7:**
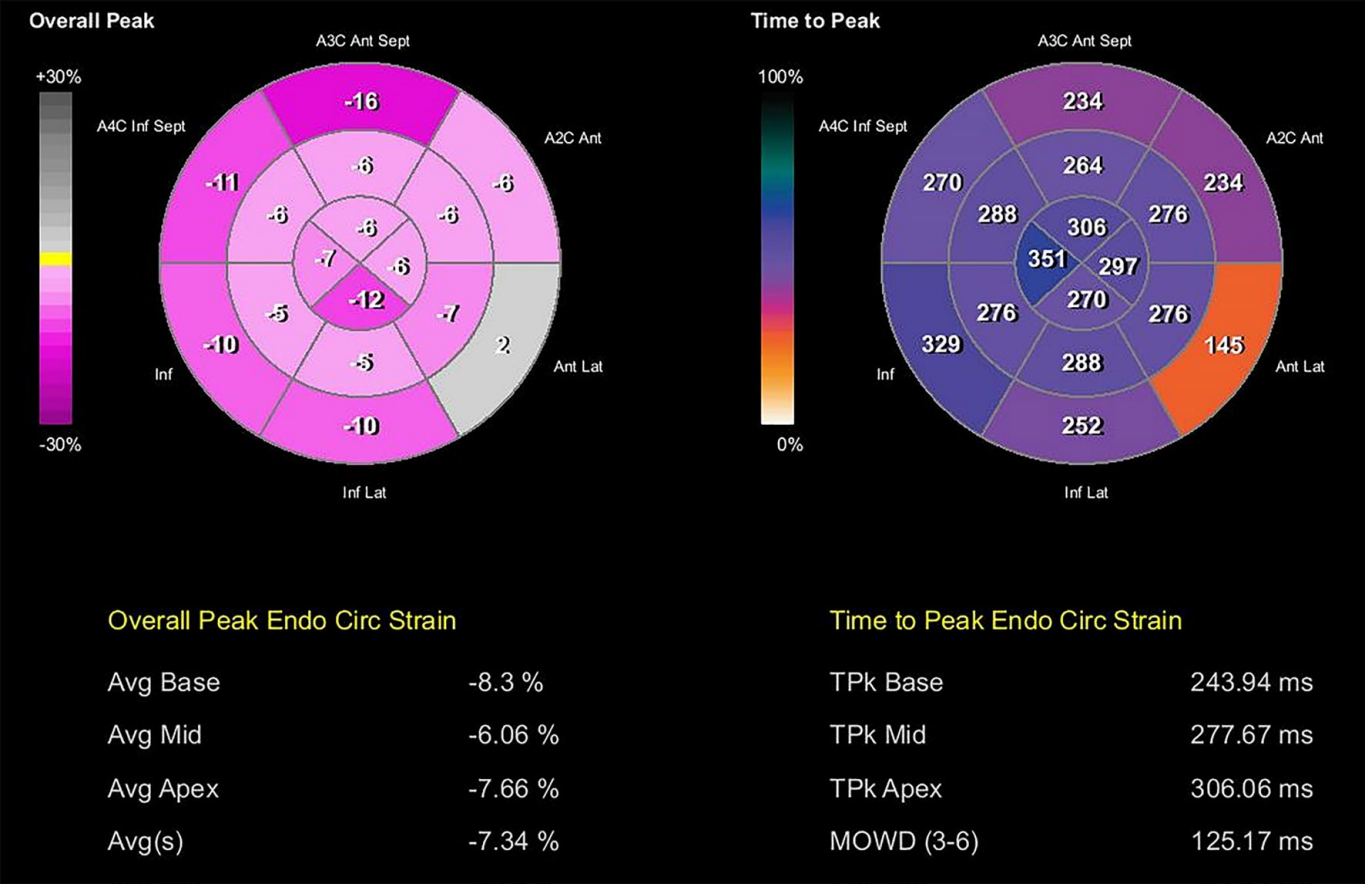
Bull’s eye plot of left ventricular circumferential strain and time to peak circumferential strain

Speckle-tracking longitudinal, circumferential, radial and transverse atrial and ventricular strain imaging was performed using velocity vector imaging (VVI) of Siemens Accuson SC 2000 (Siemens Medical Solutions USA, Inc.) (Figs. 4, 5, 6, 7, Additional files 1, 2, 3, 4, 5, 6, 7, 8, and 9: Movie clips 1–9). Compared with normal pediatric values, left ventricular longitudinal, transverse, circumferential, and radial strain were decreased [5–8]. Furthermore, there was a prestretch and rebound stretch pattern in the longitudinal strain of the left ventricle. Notably, apical rocking was detected in all four heart chambers with the direction of the apical movement in LV opposite to the rocking in the right ventricle, right atrium, and left atrium (Additional files 1, 2, 3, 4: Movie Clips 1 to 4). The results of the speckle-tracking strain imaging are depicted in Table 1.

**Table 1:** Results of speckle-tracking strain imaging of the atria and ventricles

Parameters	Right atrium	Left atrium	Right ventricle	Left ventricle
Ejection fraction %	49%	65%	–	20%
End-diastolic volume (mL)	13	13.2	–	103
End-systolic volume (mL)	6.4	4.5	–	82
Fractional area change (%)	–	–	54.2	17.9
Maximal area (cm2)	–	–	3.2	23.2
Minimal area (cm2)	–	–	1.4	19
Peak global longitudinal strain (%)	− 60%	− 10%	–	− 6.60
Average of peak segmental transverse myocardial strain ± standard deviation (SD)	− 22.50 ± 6.84	− 23.50 ± 6.27	30.5 ± 50.36	15.6 ± 5.57
Average of peak segmental longitudinal myocardial strain ± SD	12.60 ± 5.31	11.40 ± 2.48	− 5.50 ± 15.19	− 7.10 ± 3.57
Average of peak segmental radial strain ± SD	–	–	–	6.8 ± 6.75
Average of peak segmental myocardial circumferential strain ± SD	–	–	–	− 5.80 ± 1.87
Average of peak segmental transverse myocardial strain rate ± SD	2.4 ± 1.39	2.50 ± 1.07	1.70 ± 1.56	0.90 ± 0.22
Average of peak segmental longitudinal myocardial strain rate ± SD	− 0.80 ± 0.26	− 1.10 ± 0.36	− 1.20 ± 0.36	− 0.50 ± 0.20
Average of peak segmental radial myocardial strain rate ± SD	–	–	–	0.60 ± 0.22
Average of peak segmental circumferential myocardial strain rate ± SD	–	–	–	− 0.40 ± 0.16
Average of time to peak segmental myocardial transverse strain ± SD	–	–	227.80 ± 92.27	329 ± 76.28
Average of time to peak segmental myocardial longitudinal strain ± SD	–	–	266.70 ± 88.65	349.30 ± 88.38
Average of time to peak segmental radial strain ± SD	–	–	–	284.70 ± 87.41
Average of time to peak segmental myocardial circumferential strain ± SD	–	–	–	254.80 ± 41.86
Maximal opposing wall delay for transverse strain	0	18	161	125
Maximal opposing wall delay for longitudinal strain	0	35	232	71
Maximal opposing wall delay for transverse strain rate	36	36	196	36
Maximal opposing wall delay for longitudinal strain rate	107	0	304	107
Apical rocking -epicardial layer	+ 0.39	+ 0.28	–	− 0.05
Apical rocking -myocardial layer	+ 0.61	+ 0.12	+ 0.84	− 0.23
Apical rocking -endocardial layer	+ 0.72	+ 0.22	+ 0.29	− 0.17

According to the classification of Singh et al. for categorization of LV diastolic function based on the left atrial strain imaging, the patient had grade 3 LV diastolic dysfunction [9].

Given the past medical history of confirmed COVID-19 infection in the past months and normal echocardiogram in the last year, the clinical diagnosis of silent and undiagnosed COVID-19 myocarditis was suspected. COVID-19 reverse transcription-polymerase chain reaction (RT-PCR) and SARS-CoV-2 IgG and IgM antibodies were negative. Similarly, the polymerase chain reaction for other viruses, including adenovirus, enterovirus, Coxsackievirus, Epstein-Barr virus, Influenza virus, Cytomegalovirus, Human Immunodeficiency Virus, Hepatitis B and C virus, Herpes viruses were negative. The level of troponin I was normal, but NT-proBNP was elevated.

**Fig. 8:**
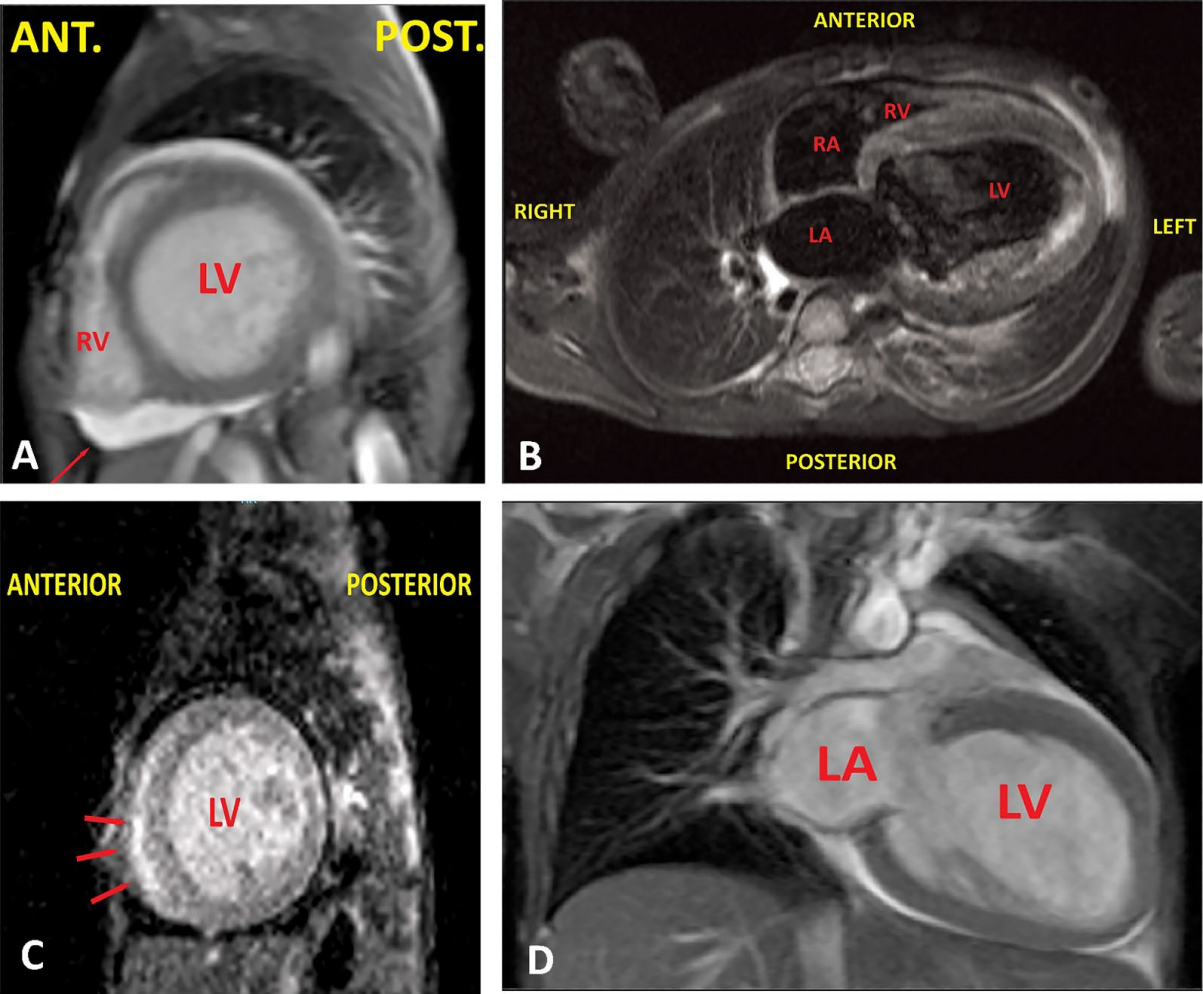
Cardiac magnetic resonance imaging of the patient. **A** Pericardial effusion, **B** indicates diffuse myocardial edema, **C** depicts late gadolinium enhancement, and **D** shows a severe enlargement of the left ventricle

To look for Lake Louise criteria of myocarditis, cardiac magnetic resonance (CMR) imaging was performed with gadolinium study and Short Tau Inversion Recovery (STIR)/T2 weighted-sequences [10]. This evaluation revealed mild pericardial effusion, severely enlarged left ventricle without left ventricular hypertrophy, evidence of diffuse myocardial edema, and severely reduced systolic function. Left ventricular and right ventricular ejection fractions were 13% and 45%, respectively, on cardiac MRI. Late-gadolinium enhancement was observed in the left ventricle's basal and mid-posterolateral segments in favor of post-myocarditis scarring (Fig. 8).

Complete workup including endocrinologic, nephrologic, neurologic, and rheumatologic causes of hypertension was performed. The only abnormal finding was significantly elevated renin (> 550 micro IU/mL, normal levels at supine position: 4.2–59.7 micro IU/mL). Measurement of aldosterone and angiotensin II was not available. Kidney function and Doppler ultrasound of renal arteries and veins were normal. Cortisol and ACTH were normal. The absence of left ventricular hypertrophy on CMR and echocardiography, absence of hypertension in the past medical history of the patient, and negative workup for other causes of hypertension were in favor of COVID-19-induced newly developed systemic hypertension.

Intravenous infusion of Dopamine (7.5 µg/kg/min), dobutamine (7.5 µg/kg/min), and milrinone (0.6 µg/kg/min), in conjunction with aspirin at a dose of 5 mg/kg/day, were administered for the patient. The patient’s hypertension was controlled by the administration of captopril (6 mg/kg/day every 6 h) and hydralazine (0.2 mg/kg/dose every 4 h). The patient left ventricular systolic function did not improve after 2 weeks of intravenous inotropic support. Therefore, the child was transferred to another tertiary center with extracorporeal membrane oxygenation and pediatric cardiac transplantation facilities.

## Discussion

This is the first report of silent myocarditis and newly developed systemic hypertension after SARS-CoV-2 infection in a child.

Silent myocarditis has been reported in several diseases such as Takayasu arteritis, myasthenia gravis, and systemic sclerosis [11–13]. However, there is no report of silent myocarditis following viral infections, let alone COVID-19 disease, to the best of our knowledge.

COVID-19 infection in children is often milder than in adults [14]. Fever and cough are the most common manifestations in children aged 0–19 years [15]. In the meta-analysis of 9335 COVID-19 patients ≤ 19 years, 13% (as mean proportion) of children were asymptomatic [15]. The risk for the development of myocarditis during SARS-CoV-2 infection has been reported to be 0.146% [16].

Furthermore, the similarity of pulmonary and cardiac involvement manifestations makes distinguishing the cardiac from the pulmonary causes challenging. The associated fever and cough in this child and the child's young age that prevents communication of symptoms further distract the primary care physician of attention to the cardiac problem.

Moreover, while Chen et al. [4] reported newly developed hypertension as a consequence of SARS-CoV-2 infection in about 8% of the 190 patients, aged 40–86 years, this sequelae has not been reported in children before. The absence of left ventricular hypertrophy, the patient's past medical history, and the completely negative workup for cardiologic, endocrinologic, nephrologic, and neurologic causes of systemic hypertension and the significantly elevated renin level were in favor of COVID-19-induced systemic hypertension (CISH). Similar to this study, renin was elevated in our patient. This newly developed hypertension may be due to increased signaling of angiotensin II by SARS-CoV-2 [4]. Genetic characteristics may play a role in various clinical features of COVID-19 infection [17, 18].

Although we performed a comprehensive strain analysis including longitudinal, circumferential, radial, and transverse strain imaging in this patient, transverse and radial strains are reported to be less reliable [5, 7].

The presence of four-chamber-apical rocking with opposite direction of rocking in the failed left ventricle compared to the three other cardiac chambers has not been reported before. Apical rocking or transverse motion of the apex is recognized as a marker of left ventricular dyssynchrony. It has been reported as a parameter that predicts response to cardiac resynchronization therapy [19–22]. We may speculate that all three other chambers are working together to compensate for abnormal left ventricular apical rocking.

Moreover, his child had prestretch and rebound stretch in the left ventricular longitudinal strain imaging. Gorcsan et al., in a study on 422 patients with heart failure, reported the presence of systolic stretch as an indicator of favorable response to cardiac resynchronization therapy in patients with a QRS duration of 120 149 ms or absent left bundle branch block [23]. Thus, the septal flash, apical rocking, and the peculiar strain pattern in this patient may predict a potential favorable response to CRT [22].

Evaluation of left atrial strain is a necessity in patients with heart failure. It provides information regarding the left atrium's reservoir, conduit, and booster function and is considered a parameter for assessing left ventricular diastolic function. Left atrial reservoir strain of less than 23% is abnormal [9, 24–28].

Cardiac magnetic resonance imaging is a robust gold standard tool for diagnosis, follow-up, and prognostication of myocarditis in acute, chronic, and healed states [29, 30]. The diagnosis of definite myocarditis was established for this patient by fulfilling the two major (myocardial edema and myocardial injury) and the two minor (pericarditis and left ventricular systolic dysfunction) criteria of the updated Lake Louise criteria [10]. Georgiopoulos and colleagues reported that late gadolinium enhancement (LGE) and anteroseptal location portend a worse prognosis in patients with acute myocarditis [31]. Myocardial edema may be seen in acute and chronic myocarditis. However, the scar is a feature of healed myocarditis [10]. Furthermore, persistent myocardial edema has been reported in 25% of patients with COVID-19 myocarditis [1].

## Conclusions

This is the first case report that indicates COVID-19 can induce silent myocarditis with progression to dilated cardiomyopathy and newly developed systemic hypertension. Accordingly, this case encompasses several critical lessons for primary care physicians and pediatric cardiologists. First and foremost, a thorough examination of the heart and measurement of blood pressure are mandatory in every child with COVID-19 infection. Second, the role of cardiac MR with appropriate sequences in the diagnosis, follow-up, and prognostication of myocarditis should not be underestimated. Third, four-chamber speckle tracking strain imaging showed apical rocking in all the four chambers of the heart in this child with secondary dilated cardiomyopathy with opposite direction in the failed left ventricle compared with other cardiac chambers. Fourth, the presence of septal flash on M-mode echocardiography, apical rocking, and prestretch–rebound stretch patterns on longitudinal strain imaging of the left ventricle may be of predictive value for response to CRT in these patients.

## Supplementary Information

**Additional file 1**. Right atrial segmental myocardial transverse and longitudinal strain imaging. As shown in the clip, apical rocking is + 0.61.

**Additional file 2**. Left atrial segmental myocardial transverse and longitudinal strain imaging. As shown in the clip, apical rocking is + 0.12.

**Additional file 3**. The right ventricular segmental myocardial transverse and longitudinal strain imaging. As shown in the clip, apical rocking is + 0.84.

**Additional file 4**. The left ventricular segmental myocardial transverse and longitudinal strain imaging. As shown in the clip, apical rocking is - 0.23.

**Additional file 5**. The right atrial global and segmental ejection fraction and segmental volume changes.

**Additional file 6**. The left atrial global and segmental ejection fraction and segmental volume changes.

**Additional file 7**. The left ventricular global and segmental ejection fraction and segmental volume changes.

**Additional file 8**. The right ventricular fractional area change.

**Additional file 9**. The left ventricular fractional area change.

## Data Availability

The datasets used and/or analyzed during the current study are available from the corresponding author on reasonable request.

## Abbreviations

CMR: Cardiac magnetic resonance imaging
CRT: Cardiac resynchronization therapy
LA: Left atrium
LV: Left ventricle
RA: Right atrium
RV: Right ventricle
